# Case Report: Articular Gout in Four Dogs and One Cat

**DOI:** 10.3389/fvets.2022.752774

**Published:** 2022-04-26

**Authors:** Hyo-Sung Kim, Hyun-Jeong Hwang, Han-Jun Kim, Sun Hee Do

**Affiliations:** ^1^Department of Veterinary Clinical Pathology, College of Veterinary Medicine, Konkuk University, Seoul, South Korea; ^2^Department of Veterinary Clinical Pathology, Konkuk Veterinary Medical Teaching Hospital, Konkuk University, Seoul, South Korea; ^3^Terasaki Institute for Biomedical Innovation, Los Angeles, CA, United States

**Keywords:** case report, monosodium urate crystals, articular gout, lameness, idiopathic polyarthritis, synovial fluid, negative birefringence, polarized microscopy

## Abstract

**Background:**

There is widespread prejudice in veterinary medicine that gout does not occur in non-human mammalians. However, we recently discovered monosodium urate crystals in the synovial fluid obtained from a few dogs and a cat. Since it is the definitive and gold standard to diagnose gout, we report these cases as newly emerging diseases in companion animals.

**Case Presentation:**

Four dogs and one cat were presented at our hospital because of lameness due to an unknown cause. Even after the routine examinations, including radiographic imaging, laboratory examination, and arthrocentesis, we were unable to find a clear cause of polyarthritis. However, we later discovered monosodium urate crystals in the synovial fluid of the animals, confirmed by polarized microscopy. In one of the two dogs treated with immunosuppressants, the disease relapsed, and the other did not show any symptoms for 3 months. The other two dogs were treated with xanthine oxidase inhibitor, where one died, and the other did not show any symptoms for 3 years. The cat was treated with drainage and intra-articular dexamethasone injection, but the disease recurred after 6 months.

**Conclusion:**

This is the first report to confirm that articular gout can occur in dogs and cats. Care must be taken not to neglect needle-shaped materials in the synovial fluid. Gout should also be included in the differential diagnosis of arthritis and further research is needed in these animals.

## Introduction

There are more than 100 joint diseases in humans, and the three most common ones are osteoarthritis, also known as degenerative arthritis; rheumatoid arthritis, which is characterized by inflammation of the synovium; and gout, where deposition of uric acid crystals in the synovium leads to immune and physical reactions (1). Gout is a metabolic and inflammatory disease whose causes, mechanisms, and treatments have long been identified in humans (2). Monosodium urate (MSU) crystals, the less-soluble crystal form of urate, are the causative agent of gout (3). These crystals can be formed when the body fluid's temperature and pH are lowered to MSU saturation (4). After growth, the crystals promote various inflammatory cascades and arachidonic acid metabolism, resulting in extreme joint pain (4). Gout was initially regarded as rheumatism, which was then distinguished by Sydenham in 1683 (2). Eighty years later, McCarty and Hollander used polarized light microscopy to differentiate it from pseudogout, whose causative crystals show positive birefringence while MSU crystals show negative (2). This method is still the gold standard for diagnosis (2, 5, 6).

Non-human mammalians, including dogs and cats, are known to have urate oxidase (uricase) which catalyzes uric acid into a soluble form. Therefore, these animals seem to be unaffected by uric acid crystals excluding conditions when these crystals lead to urolithiasis (2, 7). Although a gout-like disease was reported in animals by Miller and Kind (8), and Watson (9), these were only tentative diagnoses, without any confirmation.

To the authors' best knowledge, there have been no other reports of naturally occurring articular gout with confirmation of MSU using polarized microscopy in dogs and cats. However, we recently discovered suspicious materials in the synovial fluid of companion animals. In this report, we aimed to describe these cases confirmed to have articular gout using polarized microscopy, in addition to comparing the findings with those in humans.

## Case Description

**Figure 1:**
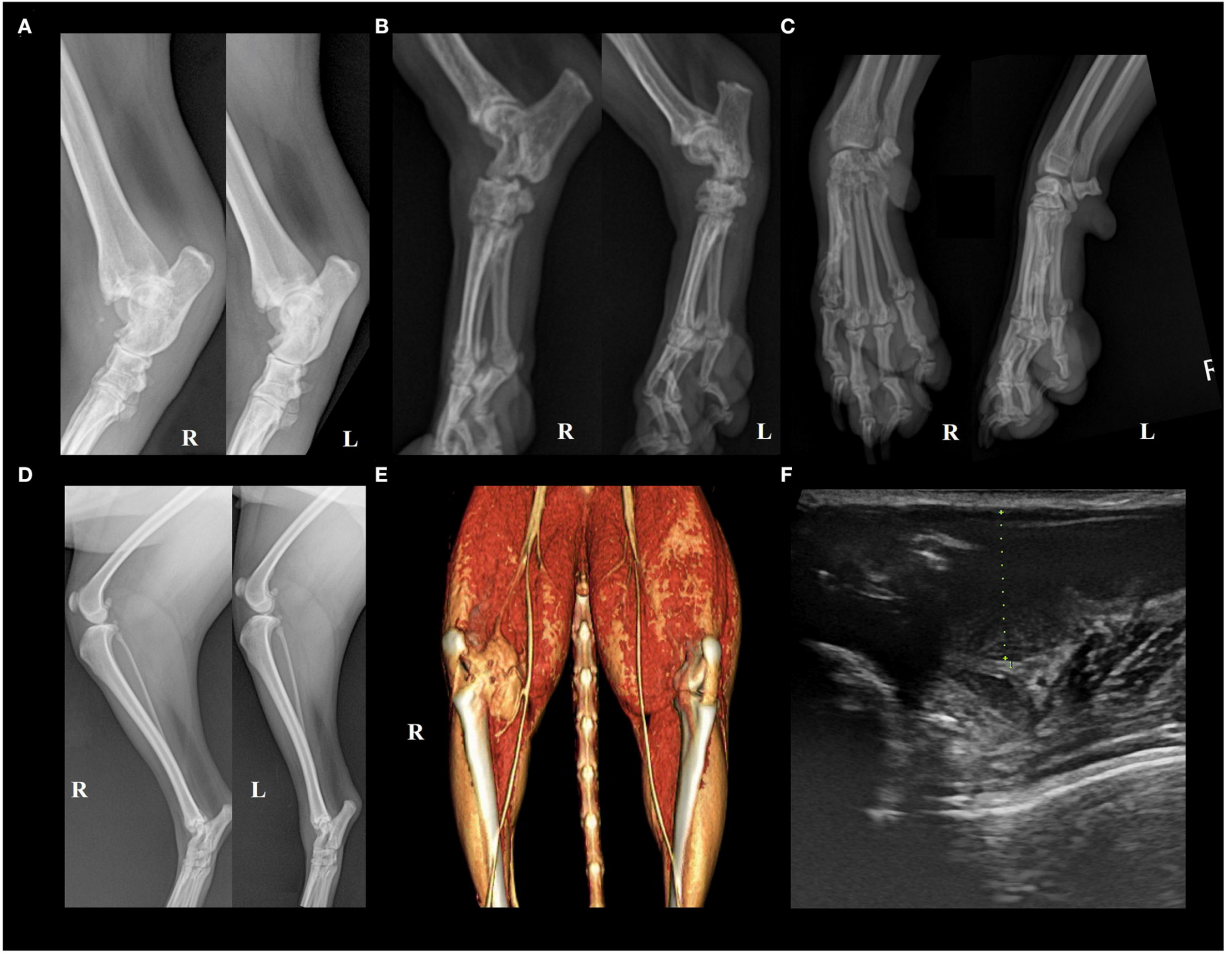
Appearance of articular gout on radiology. **(A)** Case 1. Erosive changes were seen in the right distal tibia and fibula, 4th tarsal bone, and bilateral talus. The right side limb appeared to be more severely damaged. **(B)** Case 3. Erosive changes and periosteal reactions were seen in the calcaneus, talus, and tarsal bones in bilateral tarsal joints. Overall, the bones composing the hind limb showed reduced opacity. **(C)** Case 4. Erosive changes of juxta-articular bones of left carpal joint metacarpal bone, and phalanges. Left oblique (left) and right oblique (right) views. **(D)** Case 2. Increased soft tissue opacity and thickness on the left tarsal joint region, and right infrapatellar fat pad region, compared to the contralateral side. **(E)** Case 5. 3D rendered computed tomography image showed an irregular cystic mass continuous with the right stifle joint. **(F)** Case 5. Ultrasonographic image of the cyst. Green dots indicate cystic structure, and two hyperechoic lines can be seen at the bottom of the image, mimicking the double-contour sign. Echogenic materials can be seen in the articular space.

Four dogs and one cat were presented at our hospital because of lameness due to an unknown cause. The characteristic observations of each case are summarized in Table 1. Although the degree of lameness was diverse, the dogs commonly exhibited pain, warmth, and swelling in multiple joints in physical examinations. Through orthopedic and neurological examinations, ligament damage such as collateral ligament and neurological causes were ruled out. In comparison, the cat only showed symptoms on the right stifle joint where a cystic mass recurred; this mass was resected and diagnosed as cellulitis 3 years prior (Supplementary Figure 1). On radiographic imaging, erosive changes were noted in the juxta-articular bones in dogs 1, 3, and 4 (Figures 1A–C). Osteophytes were also observed in dogs 1, 3, and 4; however, diseases such as osteochondritis dissecans were radiologically less likely, and the cause of polyarthritis could not be identified. In dog 2, however, we could not find any signs except for increased soft tissue opacity and thickness, which is only an indication of swelling (Figure 1D). In case of the cat, there was an increase in soft tissue opacity, and osteophytes were found on the juxta-articular bones (data not shown). The cystic mass seemed to be continuous with the stifle joint on computed tomography imaging (Figure 1E). On ultrasonography, it exhibited irregular margins and contained intraluminal echogenic material (Figure 1F). Despite the disease severity, the four dogs only had a mild inflammatory response with elevated C-reactive protein. Dog 2 had elevated neutrophil count as well; however, the cat did not have any remarkable changes in complete blood count and serum chemistries (Supplementary Table 1).

**Table 1:** Summary of the findings in all five cases.

**Number**	**Case 1**	**Case 2**	**Case 3**	**Case 4**	**Case 5**
Breed	Chow Chow	Pomeranian	Shih-Tzu	Beagle	Russian Blue
Age	9 years	2 years	11 years	8 years	6 years
Sex	Spayed female	Female	Spayed female	Spayed female	Spayed female
BW (BCS)	23.0 kg (7/9)	4.2 kg (6/9)	7.6 kg (7/9)	15.8 kg (7/9)	3.3 kg (4/9)
Chief complaint	Lameness of forelimb and hindlimb	Shifting lameness of forelimb Intermittent lameness of hindlimb	Shifting lameness of both hindlimbs	Intermittent lameness of left forelimb	Mass on right stifle joint, intermittent lameness of right hindlimb
Duration^1^	6 months	1 week	8 months	1 month	3 years
Lameness grade^2^	3/5	2/5	5/5	4/5	1/5
Surgical history	Bilateral patellar luxation reduction	PDA ligation	Bilateral mastectomy	TPLO	Fracture reduction Cellulitis resection
Diet^3^	Dry dog food, capelin^p^, deer ribs^p^, lamb shank bone, duck neck bone	Beef liver powder^p^, beef liver^p^, boiled chicken^p^, roasted sweet potato^f^, apple^f^, pear^f^	Dry dog food, canned dog food, meat^p^	Dry dog food mixed with pork^p^ and chicken breast^p^	Dry cat food
Localization^4^	Bilateral carpal, stifle^R^, and tarsal J	Bilateral carpal^L^, stifle^C^, and tarsal J	Bilateral shoulder, hip, stifle, tarsal^L^ J	Lt. carpal J^L^, Lt. phalanges	Rt stifle joint^R^
Radiology	Erosive changes Osteophytes Radiopaque material	UB sludge, GB sludge	Erosive change Osteophytes Osteopenia	Erosive changes Osteophytes	Osteophyte Radiopaque material Echogenic materials
Laboratory examination^5^	Total protein Globulin CRP	Neutrophil Globulin CRP	Total protein Globulin CRP (URL)	CRP	NRF
Synovial fluid	MSU crystals Large MNCs	MSU crystals Large MNCs	MSU crystals Large/small MNCs	MSU crystals	MSU crystals Neutrophils
Laboratory test	Negative for ANA, RF, Microorganisms	Negative for ANA, RF, Microorganisms	Negative for ANA, RF, Microorganisms	Negative for ANA, RF, Microorganisms	*Staphylococcus aureus* in cyst fluid
Treatment	Prednisolone Cyclosporine	Prednisolone Cyclosporine	Prednisolone Cyclosporine Allopurinol	Allopurinol Firocoxib	Drainage Enrofloxacin Dexamethasone
Prognosis^6^	Resolved in 6 weeks No symptoms for 3 months	Resolved in 8 weeks Recurred after 1 year	Died after 3 weeks	Resolved in 4 weeks No symptoms for 3 years	Repeated recurrence for 6 months

*ANA, antinuclear antibody; BCS, body condition score; BW, body weight; CRP, C-reactive protein; GB, gallbladder; MNC, mononuclear cell; MSU, monosodium urate; PDA, patent ductus arteriosus; RF, rheumatoid factor; TPLO, tibial plateau leveling osteotomy; UB, urinary bladder; URL, upper reference limit*.

1 *The time between the discovery of the symptoms and the visit to our hospital*.

2 *Grade 1, intermittent lameness; Grade 2, weight bearing, mild lameness; Grade 3, weight bearing lameness leading to gait abnormality; Grade 4, intermittent non-weight bearing lameness; Grade 5, non-weight bearing when standing*.

3 *Superscripted p indicates purine-rich diets; superscripted f indicates fructose-rich diets*.

4 *The area where most severe symptoms appeared was marked in the superscript with direction, L for left and R for right. Superscript of C indicates the joint site of cytologically evaluated synovial fluid, otherwise consistent with a superscript of direction*.

5 *Only increased results were listed*.

6 *The period after which symptoms almost disappear from treatment and the prognosis after that*.

**Figure 2:**
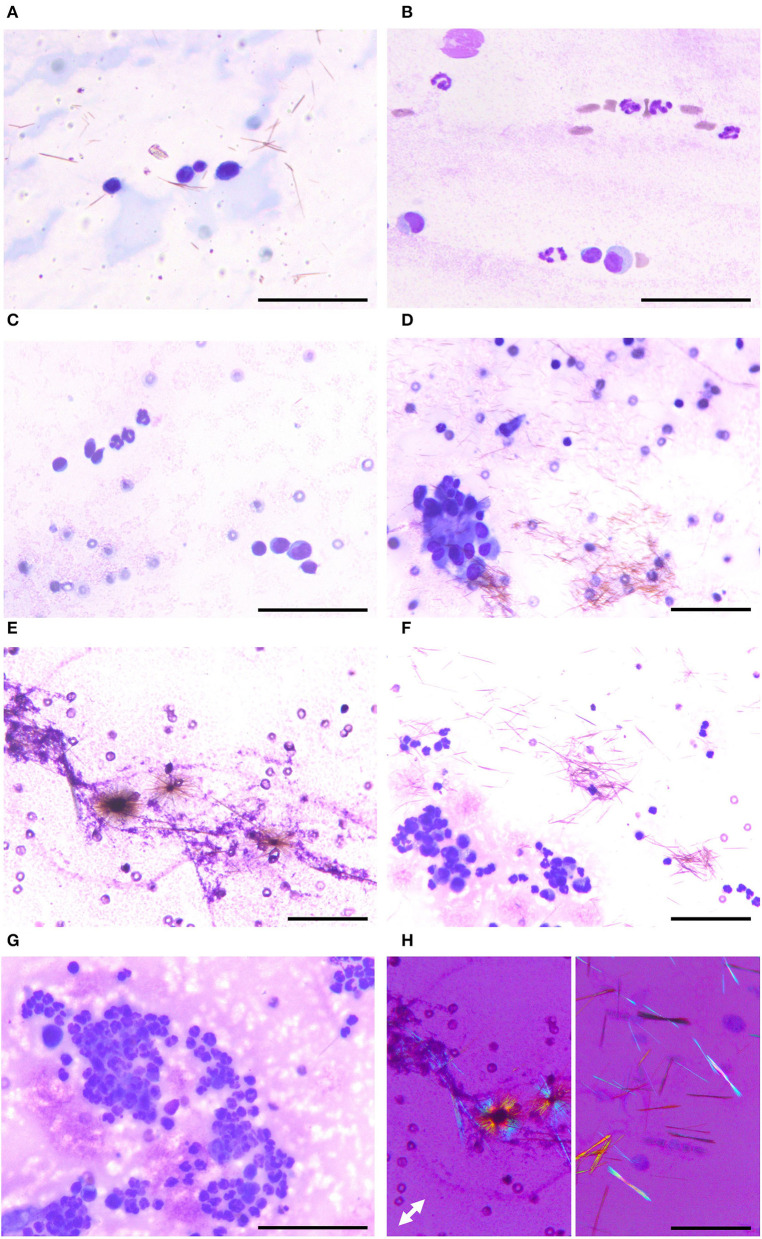
Various appearances of articular gout on synovial fluid smear cytology. **(A)** Case 1. Smear slide of the right stifle joint synovial fluid. Three large mononuclear cells and one small mononuclear cell are seen with numerous monosodium urate crystals. **(B)** Case 2. Smear slide of the right stifle joint synovial fluid. Two large mononuclear cells, one small mononuclear cell, and several neutrophils are seen. **(C,D)** Case 3. Smear slide of left tarsal joint synovial fluid. Both small and large mononuclear cells and three neutrophils are seen with aggregation of large mononuclear cells. **(E)** Case 4. Smear slide of left carpal joint synovial fluid. Monosodium urate crystals were admixed with hyaluronic acid granules, the characteristic feature of synovial fluid. **(F,G)** Case 5. Smear slide of fluid obtained from the cystic structure. Many neutrophils and several mononuclear cells are seen with monosodium urate crystals. **(H)** Needle-shaped material showing characteristic negative birefringence of monosodium urate on polarized microscopy, obtained from case 2 (left) and case 4 (right). **(A–G)** Diff-Quik staining; **(F)** polarized microscopy; white double arrow indicating slow axis; bars: 50 μm.

To identify the cause of polyarthritis in the four dogs and the cystic mass in the cat, arthrocentesis was indicated. The synovial fluid from the four dogs and the cat was clear and colorless, with a slight yellow tinge. Because the patients were small breed, fluid volume sampled was limited to <1 ml, and therefore only cytologic examination and bacterial culture were indicated. Microscopically, a coarsely granular background was observed, which indicated the hyaluronic acid-rich characteristic of synovial fluids. This was accompanied by brown, needle-shaped debris. The total nucleated cell count (TNCC) was below 3000 cells/μL or 3 cells/HPF in the four dogs. The cells were mainly large and mononuclear in dogs 1 (Figure 2A) and 2 (Figure 2B), with few neutrophils seen in the second (Figure 2B). Both large and small mononuclear cells were seen in dog 3, with a few neutrophils (Figure 2C), and aggregation large mononuclear cells (Figure 2D). In dog 4, however, cellular components were not analyzed as the few white blood cells were presumed to result from blood contamination during the procedure (Figure 2E). The synovial fluid of the cat was also composed of both small and large mononuclear cells. However, the TNCC of the cystic fluid was 46 × 10^3^/μL, mainly composed of neutrophils (Figures 2F,G). Based on these findings, immune-mediated polyarthritis and septic arthritis were considered in the differential diagnosis.

Laboratory testing for antinuclear antibody (ANA) and rheumatoid factor (RF) were negative and no organisms were cultured from the synovial fluid of the four dogs, be it fungi, aerobic, or anaerobic bacteria. Therefore, the tentative diagnosis of immune-mediated polyarthritis was made (10–12) with the exclusion of septic causes, and immunosuppressive and anti-inflammatory therapies were initiated since the sensitivity of RF test is low (13).

Dog 1 was treated with prednisolone (0.5 mg/kg, BID, PO) and cyclosporine (5 mg/kg, SID, PO). After 6 weeks, the symptoms resolved and did not recur for 3 months. The symptoms of dog 2, however, had been exacerbated despite the 2-week treatment with non-steroidal anti-inflammatory drugs (NSAIDs) (carprofen 2.2 mg/kg, BID, PO). Immunosuppressive therapy was then initiated with prednisolone (1 mg/kg, BID, PO) and cyclosporine (5 mg/kg, BID, PO). Even though the symptoms reduced and worsened repeatedly, they resolved after 7 weeks of immunosuppressive therapy. The treatment was continued by tapering the dose, but mild warmth and swelling were observed in the joints after another 6 weeks. Moreover, side effects such as pruritus and dermatitis occurred, so the prednisolone was changed to tacrolimus (0.05 mg/kg, BID, PO), but eventually, the lameness recurred after 1 year (Supplementary Figure 2). Dog 3 was also treated with prednisolone (1 mg/kg, SID, PO) and cyclosporine (5 mg/kg, BID, PO). Although the initial symptoms markedly resolved after 2 weeks, polyuria and polydipsia developed. Moreover, alanine aminotransferase, aspartate aminotransferase, alkaline phosphatase, and gamma-glutamyl transferase were increased to 671, 113, 1,804, and 47 U/L, respectively. Therefore, lepotil (4 mg/kg, BID, PO) was prescribed in addition to ademetionine (20 mg/kg, BID, PO) and ursodiol (10 mg/kg, BID, PO). Moreover, allopurinol (10 mg/kg, SID, PO) was added because of the suspicion that the needle-shaped debris was MSU crystals, and prednisolone tapering was indicated. Unfortunately, the owner notified us that the dog died after 3 weeks. Dog 4 was also given NSAIDs (firocoxib, 5 mg/kg, SID, PO), and ademetionine (12.5 mg/kg, SID, PO). The symptoms improved after 1 month similar to the other dogs. However, the needle-shaped material was confirmed to be MSU crystals by polarized microscopy and the final diagnosis of articular gout was made. To treat the disease properly, a xanthine oxidase inhibitor (allopurinol, 10 mg/kg, SID, PO) was prescribed and a low-protein diet and weight loss were recommended. After 3 months, the symptoms completely resolved and the dog was not brought again to the hospital for 3 years.

In case of the cat, however, we excluded crystal-induced arthritis from the differential diagnosis since there were no reports of feline articular gout. We continued antibiotic therapy (enrofloxacin, 5 mg/kg, SID, PO) for 2 more weeks with side effects including ocular toxicity monitored because septic arthritis was the most suspected cause. Over the next 2 months, the lumen refilled again twice, which was treated with drainage and intra-articular dexamethasone injections (5 mg/ml, 1 ml). The cat underwent surgical cyst removal because of the recurrence, despite the subsiding inflammation measured by TNCC. After post-operative care, specific treatment for feline articular gout will be considered.

The common finding in all these case reports was the presence of needle-shaped material in the synovial fluid. They were all confirmed later as MSU crystals, showing characteristic negative birefringence on polarized microscopy (Figure 2H). Therefore, the final diagnosis of articular gout was made in all five cases. For further verification, we scraped the archived slides with MSU for scanning electron microscopy with energy-dispersive spectroscopy (SEM-EDS) analysis (Supplementary Figure 3). We confirmed that the observed crystals do not contain calcium (Ca, differentiation point from calcium pyrophosphate crystals) and have elements of MSU, carbon (C), nitrogen (N), oxygen (O), and sodium (Na).

## Discussion

### Dietary Habits and Obesity Are Likely Risk Factors for Gout

Uric acid is the final product of purine metabolism. Therefore, a purine-rich diet with meat, fish, and alcohol is traditionally known to be the main cause of gout (2, 3, 14). It has also been shown that a fructose-rich diet with fruits and processed food accelerates the catabolism of adenine nucleotide, which can be a risk factor as well (2). Other human risk factors include male sex, post-menopausal state, chronic heart failure, hypertension, poor kidney function, diuretic use, obesity, diabetes, and age (14). All dogs described in this case report had a diet rich in purine, especially dog 2 who had high fructose intake as well without any commercial diet. Unlike humans, the animals in this case report were all female, but three of the dogs and the cat had undergone ovariohysterectomy, which can be paralleled to a post-menopausal state. Dog 2 is thought to have been affected by the long-term use of diuretics and systemic hypertension due to patent ductus arteriosus surgery. Similar to the other risk factors that were previously mentioned, many animals were obese, represented by the body condition score, and all but two were middle-age to old (>6 years). These findings show that companion animals have similar risk factors as humans when it comes to gout.

### Time-Varying Symptoms Are Similar to Human Gout

All the animals in this case report were presented to our hospital with lameness as the chief complaint. Moreover, warmth, swelling, pain response, and disuse atrophy were generally observed. Such symptoms are non-specific findings and can be seen in other conditions (15). The duration of lameness, however, seems similar to that of gout in humans and experimental models. In humans, most pain occurs within 24 h, and symptoms resolve within 14 days even without treatment (16, 17). Similarly, Hassan et al. reported the symptoms to be most severe at 4 h and relieved after 24 h in the dogs when a sodium urate suspension is injected intra-articularly (18). The owners of the animals in this case report also described the symptoms as intermittent or shifting lameness, similar to the time course of symptomatic episodes that can be seen in humans. The joint location is also similar since it is more likely to occur in areas with lower local temperatures, such as the upper extremities (4).

### Erosion and Double Contour Sign Are Major Findings on Imaging

The American College of Rheumatology/European League Against Rheumatism (ACR/EULAR) introduced classification criteria to aid the diagnosis of gout by scoring clinical, laboratory, and imaging objects. According to this criterion, the individual can be classified as having gout if the total score is eight or more. Erosion of the hands and feet, seen on conventional radiograph imaging, is considered strong evidence of gout in humans, checking four points out of the eight needed (6, 19). In our case report, there was only one dog confirmed to have erosive changes of the digital bones. Unfortunately, we were unable to reinspect the other cases because such signs can be easily missed in veterinary medicine due to the restraining process. Therefore, more attention is needed to differentiate gout from other arthritis exhibiting erosive changes, especially rheumatoid arthritis.

It is more effective to use dual-energy computed tomography or magnetic resonance imaging to demonstrate urate deposits. However, high cost and accessibility issues apply in veterinary medicine. Thus, alternatives are needed (20). Ultrasonography can be another option that is relatively cheaper and effective for diagnosis (20). The ACR/EULAR has demonstrated that the double-contour sign (DCS), which is a hyperechoic irregular enhancement of the cartilage, is evidence of urate deposition (6, 21). Others include bright stippled aggregates visualized as hyperechoic foci with or without shadowing and hypervascularity visualized by power Doppler signal density (21). We found DCS-mimicking lines on the articular space in the cat in this case report. Unfortunately, ultrasonography was not used in the other cases because there was a lack of information on gout and its visual features. Therefore, it is highly recommended to pay more attention to the radiologic images in cases of idiopathic arthritis.

### Diagnosis Is Through MSU Crystal Confirmation Using Polarized Microscopy

Serum urate is a significant feature of human gout; however, it can be measured differently depending on the disease severity. Therefore, the highest serum urate level along the time course of disease is used in the ACR/EULAR's diagnostic criterion (6). However, blood uric acid was not detected in our cases. In the synovial fluid, we found various cells at first, including neutrophils, small mononuclear cells, and large mononuclear cells, even though the TNCC was below the limit. Nevertheless, we could not exclude other immune-mediated causes due to the lack of sufficient explanation for polyarthritis (15). However, we began to look for needle-shaped crystals because these various cells can be seen in both human gout and canine synovitis models induced by MSU (21, 22).

A polarized microscopy is a tool usually used to determine the properties of minerals, and various substances can exhibit birefringences, such as helminths, collagen fibers, and foreign materials (23). However, they can be easily differentiated from needle-shaped crystals by their morphology. Various needle-shaped crystals can be seen in body fluids, such as tyrosine, bilirubin, ampicillin, sulfonamide, and acyclovir. However, they are usually present in urine. Calcium pyrophosphate which causes pseudogout is also needle-shaped, although it can be seen as a tablet or a parallel piped shape as well, with weak positive birefringence (24). Cholesterol, corticosteroid, calcium oxalate, and calcium phosphate crystals can also be seen in synovial fluid. Among these crystals, only MSU is needle-shaped with negative birefringence, which means that the refractive index of perpendicular waves is smaller than that of parallel ones. For these reasons, confirming the MSU, needle-shaped, negative birefringence crystal in synovial fluid, is a definitive and sufficient criterion, wildly accepted in medicine as the gold standard for the diagnosis of gout (25).

### Treatment Options for Articular Gout in Non-human Mammals

Gout causes extreme pain, described as “pain even in the breeze” in the orient culture. It is known as a refractory disease without a cure. However, it can be managed efficiently with drugs and self-care. For acute gout, NSAIDs are recommended as first-line treatment because they have shown effectiveness in other immune-mediated arthritis (2). NSAIDs have also been proven to be effective in the crystal-induced synovitis model of dogs (26). Moreover, dogs 2 and 4 in this case report had NSAIDs treatment, and it was partial but effective, suggesting that they can be used as well in mammalians. Immunosuppressants also have some effect on gout and are used for concomitant therapy (27). Prednisolone and cyclosporine reduced the symptoms in dogs 1, 2, and 3. However, caution should be exercised in its use as long-term immunosuppressive treatment exhibits various side effects. Draining the synovial fluid and injecting lidocaine and glucocorticoids into the joint cavity can relieve the pain, but repetitive procedures can have adverse effects. In this case, the cat had recurrent cysts despite repetitive treatment, suggesting that while this method can relieve pain, it cannot be a fundamental treatment.

In human medicine, there are various medications to specifically treat gout. Colchicine is a traditionally effective drug for gout treatment, and the patient's reactivity was used for diagnosis as well (25). Although veterinary usage was reported for different purposes, colchicine may be used for mammalian gout treatment (28). Allopurinol, a xanthine oxidase inhibitor, is known to reduce uric acid concentrations in serum and urine, and it was proven to be effective in treating gout in dog 4. However, it must be administered carefully in mammals with renal or hepatic insufficiency. A prescription that did not take this into account is suspected to be the cause of dog 3's death. Like allopurinol, febuxostat, a novel non-purine selective xanthine oxidase inhibitor, is also effective on gout with less adverse effect and may be used in dogs (29). In this report, allopurinol was the only medication with proven therapeutic efficacy. However, given the similarity of the mechanisms of action, the abovementioned two medications can be suggested as possible treatment options for mammalian gout.

### Increasing Prevalence of Articular Gout in Dogs and Cats

This case report alone cannot accurately explain why articular gout has not been diagnosed previously among non-human mammalians. It may have gone unnoticed for decades, as it did in humans, or it may be due to the relatively late application of polarized microscopes and other state-of-the-art equipment. It is also possible that it is a newly emerged disease, caused by a prolonged life span and indiscriminate food abuse with a so-called “handmade, wellbeing diet,” making crystals undissolvable (4). X-linked dominant genetic mutation might have occurred in animals, just like humans (14). In order to determine the cause, more studies are needed in the future through measuring the xanthine concentration, phenotyping, and genetic analysis.

Idiopathic polyarthritis is the most common cause of polyarthritis in veterinary medicine (15, 30, 31). Therefore, we strongly believe that many mammals treated for idiopathic arthritis might actually have gout. We had additionally found five more cases with MSU crystal deposits, 2 in the articular joint, 2 in the salivary mucocele, and 1 in the abdominal fluid. However, we did not include them because of the lack of history and follow-up. It seems like there are more cases, as more dogs with MSU crystals are coming in at the time of writing this manuscript. There is a need for future research focusing on establishing diagnostic criteria and treatment protocols. For example, the ACR/EULAR announced classification criteria in 2015 to diagnose gout by scoring clinical symptoms, serum urate concentration, and imaging criteria even if arthrocentesis cannot be performed (6). However, it cannot be applied in veterinary medicine because of symptom identification difficulty and lower serum urate levels compared to humans. Therefore, it is necessary to establish a novel standard suitable for non-human mammalians.

## Concluding Remarks

In this case report, we highlighted the fact that articular gout does occur in dogs and cats. As far as we know, this is the first report to confirm it using polarized microscopy in the veterinary literature. The needle-shaped material should not be dismissed as artifacts but included in the arthritis diagnostic process. Medicine is a science aimed at diagnosing, treating, and preventing diseases, and we should not practice it only with algorithms of biased experience. Many mammals have idiopathic arthritis and immunosuppressive therapy may result in overtreatment of those who actually have gout (11, 23). We have provided strong evidence through demonstrating several cases with articular gout confirmed by polarized microscopy. Frontline clinicians should decide whether to apply this case report in practice to accurately diagnose and treat these mammals, or manage them as “idiopathic arthritis.”

## Data Availability Statement

The original contributions presented in the study are included in the article/Supplementary Material, further inquiries can be directed to the corresponding author.

## Author Contributions

H-SK drafted the manuscript. H-JH and H-JK edited the manuscript. SD confirmed the cases and revised the final submission. All authors contributed to the article and approved the submitted version.

## Funding

This article was supported by Konkuk University in 2021.

## Conflict of Interest

The authors declare that the research was conducted in the absence of any commercial or financial relationships that could be construed as a potential conflict of interest.

## Publisher's Note

All claims expressed in this article are solely those of the authors and do not necessarily represent those of their affiliated organizations, or those of the publisher, the editors and the reviewers. Any product that may be evaluated in this article, or claim that may be made by its manufacturer, is not guaranteed or endorsed by the publisher.

## Supplementary Material

Supplementary Figure 1 Clinical history of the cat before referring to our hospital.

Supplementary Figure 2 Treatment schedule of dog 2.

Supplementary Figure 3 Scanning electron microscopy analysis of monosodium urate crystals. **(A)** SEM-EDS results of commercially available MSU powder. **(B)** SEM-EDS results of monosodium urate crystals scraped from slides.

Supplementary Table 1 Complete blood count and serum chemistry results.

## Abbreviations

ACR/EULAR: American College of Rheumatology/European League Against Rheumatism
ANA: antinuclear antibody
DCS: double-contour sign
MSU: monosodium urate
NSAID: non-steroidal anti-inflammatory drug
RF: rheumatoid factor
SEM-EDS: scanning electron microscopy with energy-dispersive spectroscopy
TNCC: total nucleated cell count.
