# Supplementary figures and images for: Case Report: Articular Gout in Four Dogs and One Cat
Source: Front Vet Sci. 2022 Apr 26;9:752774. doi: 10.3389/fvets.2022.752774 (PMC9087635; doi:10.3389/fvets.2022.752774)

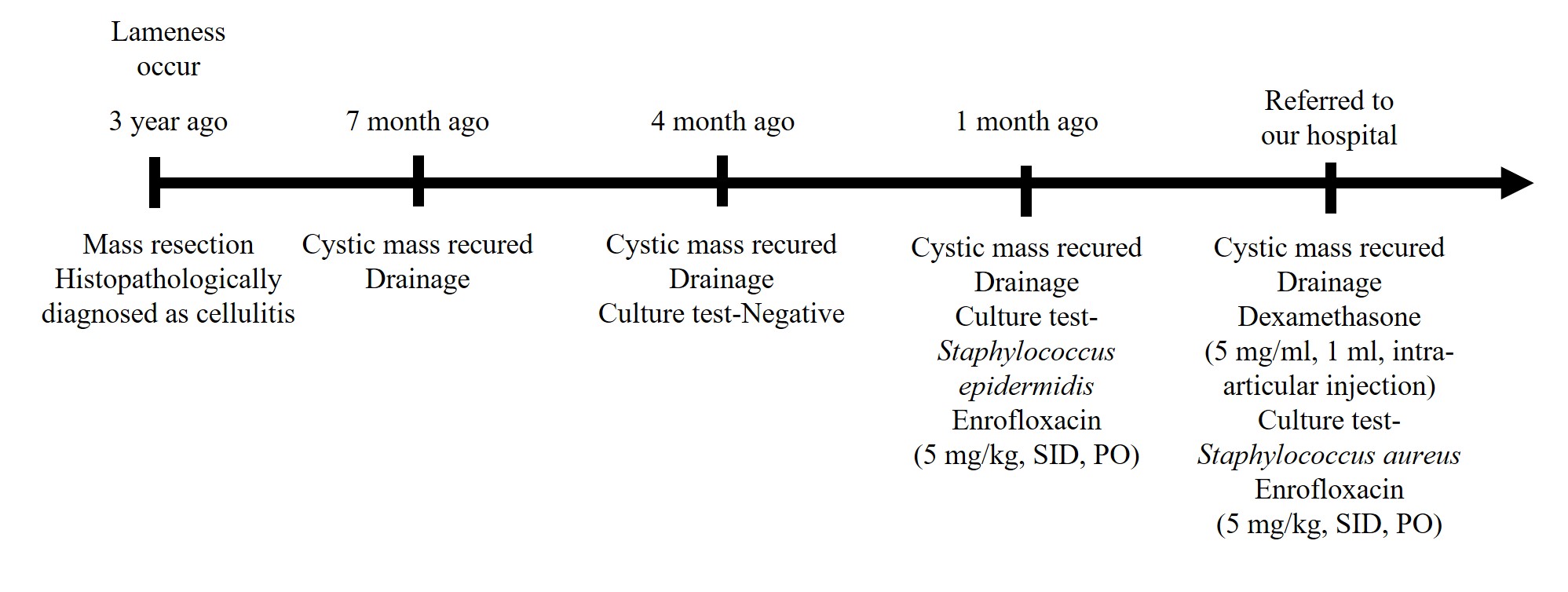

Supplement: Supplementary Figure 1 — Clinical history of the cat before referring to our hospital. [file Image_1.JPEG]

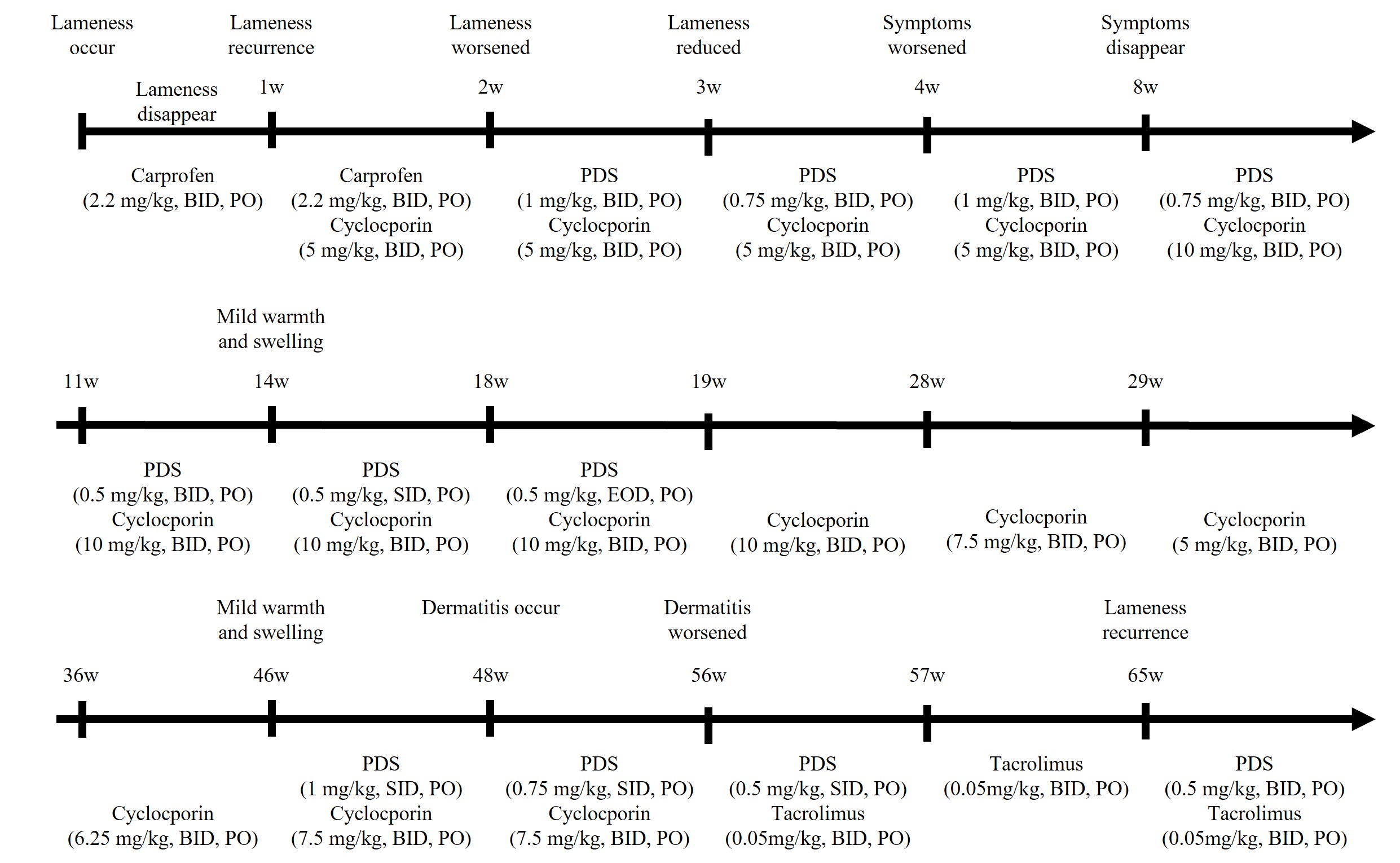

Supplement: Supplementary Figure 2 — Treatment schedule of dog 2. [file Image_2.JPEG]

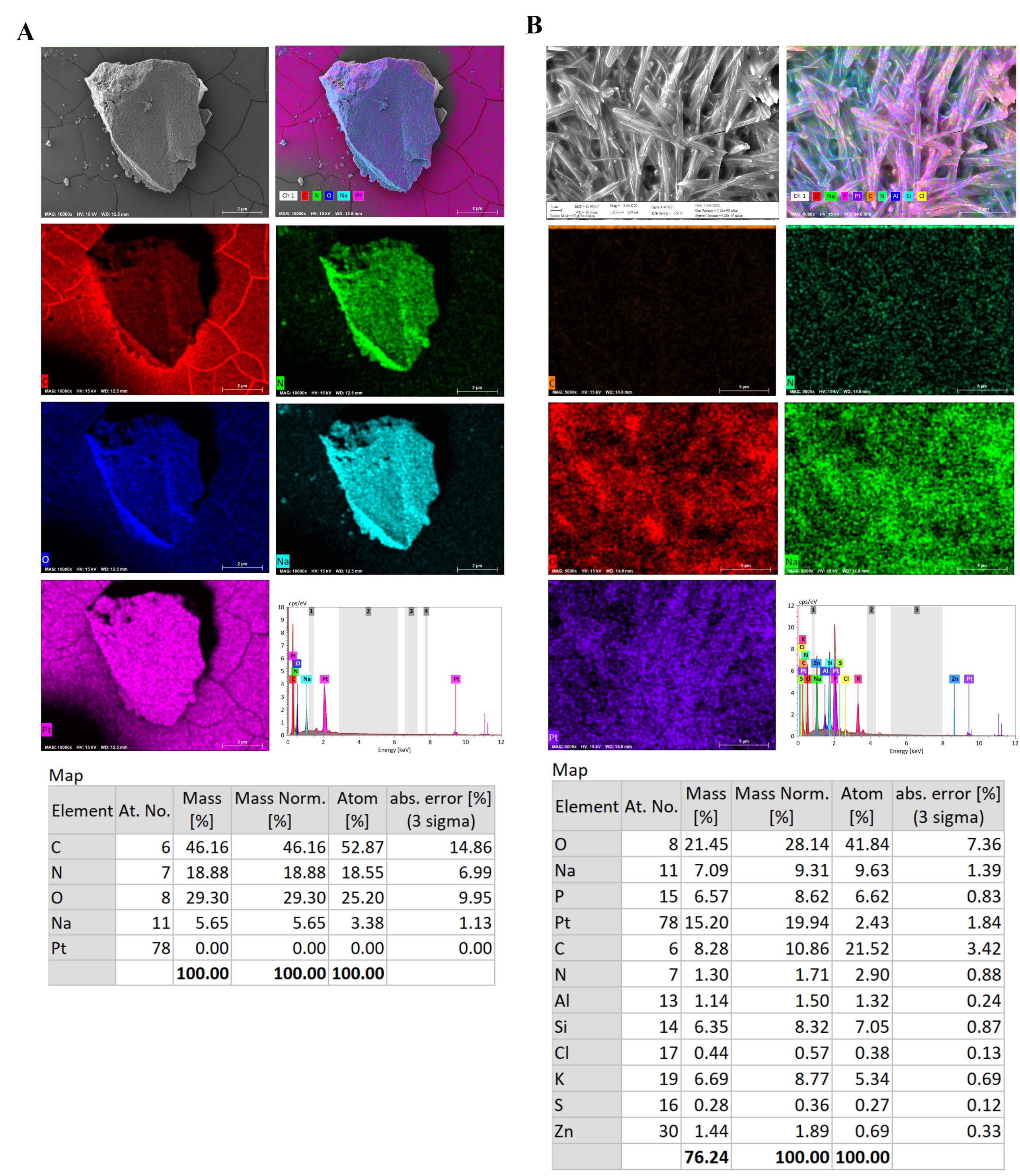

Supplement: Supplementary Figure 3 — Scanning electron microscopy analysis of monosodium urate crystals. (A) SEM-EDS results of commercially available MSU powder. (B) SEM-EDS results of monosodium urate crystals scraped from slides. [file Image_3.JPEG]
