# Supplementary material for: Case Report: Articular Gout in Four Dogs and One Cat
Source: Front Vet Sci. 2022 Apr 26;9:752774. doi: 10.3389/fvets.2022.752774 (PMC9087635; doi:10.3389/fvets.2022.752774)
Supplement: Supplementary Table 1 — Complete blood count and serum chemistry results. [file Table_1.docx]

Supplementary Material

# Supplementary Table 1. Complete blood count and serum chemistry results.

|  | Unit | Reference range (canine) | Case 1 | Case 2 | Case 3 | Case 4 | Reference range (feline) | Case 5 |
| --- | --- | --- | --- | --- | --- | --- | --- | --- |
| RBC | 10 x 12/L | 5.65~8.87 | 5.82 | 8.79 | 8.27 | 7.08 | 6.54~12.2 | 7.22 |
| HCT | % | 37.3~61.7 | 36.4▼ | 49.9 | 45.8 | 47.4 | 30.3~52.3 | 34.5 |
| HGB | g/L | 13.1~20.5 | 122▼ | 172 | 160 | 169 | 9.8~16.2 | 11.6 |
| RETIC | K/μL | 10~110 | 30.8 | 73.8 | 27.3 | 57.3 | 3~50 | 16.6 |
| WBC | 10 x 9/L | 5.05~16.76 | 9.95 | 20.13▲ | 9.41 | 9.73 | 2.87~17.02 | 15.08 |
| NEU | K/μL | 2.95~11.64 | 6.41 | 16.37▲ | 6.64 | 7.7 | 1.48~10.29 | 11.12▲ |
| LYM | K/μL | 1.05~5.1 | 2.29 | 3.04 | 1.93 | 1.53 | 0.92~6.88 | 3.13 |
| MONO | K/μL | 0.16~1.12 | 0.49 | 0.38 | 0.55 | 0.31 | 0.05~0.67 | 0.36 |
| EOS | K/μL | 0.06~1.23 | 0.76 | 0.32 | 0.29 | 0.16 | 0.17~1.57 | 0.41 |
| BASO | K/μL | 0~0.1 | 0 | 0.02 | 0 | 0.03 | 0.01~0.26 | 0.06 |
| PLT | K/μL | 148~484 | 362 | 291 | 550▲ | 401 | 151~600 | 455 |
| Protein-Total | g/L | 52~82 | 94▲ | 79 | 87▲ | 71 | 57~89 | 79 |
| Albumin | g/L | 23~40 | 29 | 31 | 29 | 38 | 22~40 | 33 |
| Globulin | g/L | 25~45 | 65▲ | 48▲ | 58▲ | 33 | 28~51 | 46 |
| Glucose | mg/L | 700~1430 | 920 | 1380 | 1100 | 1090 | 740~1590 | 1460 |
| ALT | U/L | 10~125 | 19 | 12 | 23 | 22 | 12~130 | 18 |
| AST | U/L | 0~50 | 19 | 22 | 29 | 22 | 0~48 | 32 |
| ALP | U/L | 23~212 | 177 | 109 | 122 | 153 | 14~111 | 36 |
| SDMA | μg/L | 0~140 | 90 | 60 | 100 | 40 | 0~140 | 70 |
| BUN | mg/L | 70~270 | 70 | 110 | 70 | 140 | 160~360 | 190 |
| Creatinine | mg/L | 5~18 | 11 | 7 | 6 | 7 | 8~24 | 18 |
| BUN: Creatinine Ratio |  | 4~27 | 6 | 15 | 12 | 20 | 4~33 | 10 |
| Uric Acid | mg/L | 0~10 | Under | Under | Under | Under | 0~10 | Under |
| Creatine Kinase | U/L | 10~200 | 204▲ | 68 | 86 | 62 |  |  |
| CRP | mg/L | 0~10 | 20▲ | 93▲ | 10 | 10.96▲ |  |  |
| Lactate | mmol/L | 0.5~2.5 | 3.62▲ | 11.12▲ | 1.47 | 1.35 |  |  |
| LDH | U/L | 40~400 | 232▲ | 189▲ | 238 | 1512▲ |  |  |
| Sodium | mmol/L | 144~160 | 152 | 150 | 154 | 155 | 150~165 | 161 |
| Potassium | mmol/L | 3.5~5.8 | 4.7 | 4.7 | 4.2 | 4.3 | 3.5~5.8 | 4.8 |
| Chloride | mmol/L | 109~122 | 111 | 107▼ | 112 | 117 | 112~129 | 121 |

The increased values were highlighted with ▲ and decreased value with ▼.

ALT, alanine aminotransferase; ALP, alkaline phosphatase; AST, aspartate aminotransferase; BASO, basophil; BUN, blood urea nitrogen; CRP, C-reactive protein; EOS, eosinophil; HCT, hematocrit; HGB, hemoglobin; LDH, lactate dehydrogenase; LYM, lymphocyte; MONO, monocyte; NEU, neutrophil; PLT, platelet; RBC, red blood cell; RETIC, reticulocyte; SDMA, symmetric dimethylarginine; WBC, white blood cell;
